# Dppa2 Promotes Early Embryo Development Through Regulating PDH Expression Pattern During Zygotic Genome Activation

**DOI:** 10.3390/ijms26073436

**Published:** 2025-04-06

**Authors:** Anqi Di, Xinyi Zhang, Lishuang Song, Song Wang, Xuefei Liu, Chunling Bai, Guanghua Su, Guangpeng Li, Lei Yang

**Affiliations:** 1 State Key Laboratory of Reproductive Regulation and Breeding of Grassland Livestock, College of Life Science, Inner Mongolia University, Hohhot 010021, China; anqi_di@126.com (A.D.); zhangxinyi2356@163.com (X.Z.); xiaoshuang2000@126.com (L.S.); liuxuefei1006@126.com (X.L.); chunling1980_0@163.com (C.B.); suguanghua0707@163.com (G.S.); 2 College of Life Science, Northeast Agricultural University, Harbin 150030, China; wangsong199852@163.com

**Keywords:** zygotic genome activation, Dux, Dppa2, Dppa4, TCA, PDH

## Abstract

During embryonic development, zygotic genome activation (ZGA) is a critical event that determines the rational process and the fate of embryonic cells. The tricarboxylic acid cycle (TCA cycle) provides necessary reactants and energy for biological activities such as genome activation, chromatin opening, and epigenetic modifications during ZGA. Recent studies have shown that during ZGA, core enzymes associated with TCA briefly enter the nucleus and participate in initiating the ZGA process. However, the regulatory relationship between ZGA factors, such as Dux, Dppa2, and Dppa4, and the core enzymes of the TCA cycle remains unknown. In this study, we found that Dppa2 plays a key role in ZGA by directly determining the localization of TCA core enzymes, thereby affecting the early embryonic development. To further investigate the effect of Dppa2 on the localization of pyruvate dehydrogenase (PDH), we followed the establishment of an inducible Dppa2 transgenic mouse model. We found that the “chronoectopic” expression of Dppa2 prior to normal ZGA time could lead to the advanced nuclear localization of PDH. In summary, Dppa2 plays a key role in ZGA, directly determining the location of TCA core enzymes in early embryos. This study provides a theoretical basis for early embryonic development at the metabolic regulation level.

## 1. Introduction

The phenomenon whereby a spermatozoon makes its journey to enter and unite with an oocyte is termed fertilization. After fertilization, the zygote genome is activated (ZGA), while maternal RNA and proteins are degraded [1]. In mice, the Minor ZGA phase of transcriptional activation occurs in the late one-cell embryo stage, which initiates limited gene expression. Subsequently, the Major ZGA phase begins at the late two-cell stage and induces a significant increase in global RNA synthesis [2]. The zygote and two-cell embryo are totipotent, possessing the ability to differentiate into all kinds of cell types. Interestingly, a rare subpopulation of stem cells, referred to as “2C-like cells”, are generated during embryonic stem cell culture. These cells exhibit numerous similarities to the transcriptional and epigenetic regulatory mechanisms observed during early embryonic development. Therefore, 2C-like cells are frequently used as an in vitro analog to study the process of ZGA [3,4].

Previous studies have shown that Dux is a double homeodomain protein that acts as a critical regulator of gene activation during ZGA in 2C-like cells [5,6]. The depletion of Dux with microsatellite repeats by zygotic CRISPR/Cas9 injection showed that Dux target sites in 2C-like cells and ZGA-related genes are normally activated [7]. In addition, the absence of Dux only slightly affected ZGA, resulting in a reduction in litter sizes (6). In 2C-like cells, developmental pluripotency associated 2 and 4 (Dppa2/4) control the zygotic transcriptional program by activating Dux [8,9]. Dppa2/4 independently regulate young LINE-1 in ESCs via Dux [10]. Dppa2/4 activate transcription factors in mouse ESCs and are pluripotent markers essential for embryonic development [10,11]. However, the key factors that activate embryonic ZGA are unknown.

Taking mouse embryos as an example, when ZGA occurs during preimplantation development, significant changes occur in epigenetic modifications, including methylation, acetylation, phosphorylation, glycosylation, and others [4]. Among them, the methyl group required for methylation modification is mainly provided by S-adenosylmethionine (SAM) in TCA. In addition, acetyl coenzyme A, ATP, and UDP GlcNAc participate in protein acetylation, phosphorylation, and glycosylation, respectively, which are all generated by the TCA cycle driven by mitochondrial enzymes [12,13]. Therefore, the normal metabolism of the TCA cycle is critical in the correct modification of histones and the occurrence of ZGA.

In the present study, we used siRNA injection to knock down Dux/Dppa2/Dppa4 during preimplantation embryonic development in mice. The knockout of Dppa2 induced arrest in most of the embryos at the two-cell stage, and the blastocyst rate decreased significantly. Moreover, the knockdown of Dppa2 resulted in the mislocution of pyruvate dehydrogenase (PDH) in the nucleus. It can be inferred that Dppa2 directly determines the location of TCA core enzymes in early embryos and significantly affects embryonic development.

## 2. Results

### 2.1. Effects of Dux, Dppa2, and Dppa4 Gene Knockdown on Embryonic Development

**Figure 1:**
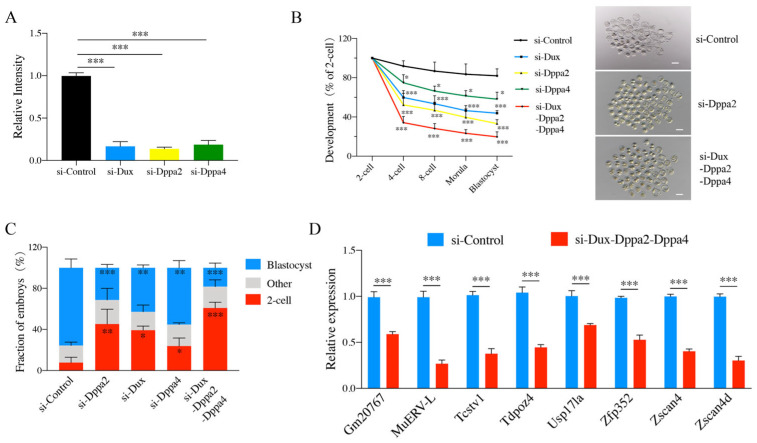
Embryo arrestment at two-cell stage after knockdown experiments. (**A**) Efficiency detection of siRNA knockdown. (**B**) Developmental rates of embryos derived using different siRNA. (**C**) Stacked bar plots show the fraction of embryos at blastocyst stages after injection with different siRNA. (**D**) Relative expression of ZGA-associated genes (MuERVL, Zscan4d, Tcstv1, Usp17la, Zscan4, Zfp352, Tdpoz4, and Gm20767) in Dux, Dppa2, and Dppa4 joint knockdown. Reference gene: Gapdh. Mean ± s.d.; n = 3 independent experiments or biological replicates. * *p* < 0.05, ** *p* < 0.01, *** *p* < 0.001, as compared with control group, by two-tailed Student’s *t*-test.

To investigate the role of Dux, Dppa2, and Dppa4 in mouse preimplantation embryos, we synthesized the corresponding small-interfering RNA (si-RNA) and injected the si-RNA into the cytoplasm of the zygotes. The results showed that the knockdown efficiencies of Dux, Dppa2, and Dppa4 were 83.12%, 89.2%, and 81.33%, respectively (Figure 1A). Then, we co-injected the three genes’ siRNAs into zygotes to analyze the possible collective impact on embryonic development. Blastocyst development declined to 19.78 ± 5.05% compared to the control value of 80.36 ± 9.81% (*p* < 0.001) (Figure 1B and Table S1). Most of the embryos injected with Dux, Dppa2, and Dppa4 siRNAs arrested at the two-cell stage (Figure 1B,C and Table S1). These findings suggest that Dux, Dppa2, and Dppa4 play an important role in embryo development. However, some embryos could still develop into blastocysts. This observation is consistent with recent studies suggesting that Dux, Dppa2, and Dppa4 are important but not indispensable for in vivo embryo development [6,7]. To further characterize the effects of individual genes on embryonic development, the individual gene knockdown of Dux, Dppa2, and Dppa4 was performed. The results were consistent with the pattern observed when all three genes were knocked down simultaneously. The knockdown of Dux, Dppa2, or Dppa4 alone also resulted in two-cell arrest and reduced blastocyst development (Figure 1B,C).

After Dux depletion, the incidence of two-cell block was 39.24 ± 4.06%, and the blastocyst rate was significantly lower than that of the control (43.81 ± 2.69% vs. 80.36 ± 9.81%, *p* < 0.01) (Figure 1B,C and Table S1). Similarly, after Dppa2 knockdown, the two-cell block rate was 45.21 ± 14.43%, and the blastocyst rate was significantly decreased (33.20 ± 4.10% vs. 80.36 ± 9.81%, *p* < 0.001) (Figure 1B,C and Table S1). The knockdown of Dppa4 resulted in a two-cell arrest rate of 23.85 ± 7.77%, and the blastocyst rate was also significantly reduced (58.15 ± 7.09% vs. 80.36 ± 9.81%, *p* < 0.05) (Figure 1B,C and Table S1). These results strongly implied that Dux, Dppa2, and Dppa4 are intimately associated with early embryonic development. We confirmed that Dux knockdown caused delayed ZGA and reduced embryo survivability [6]. Especially, the knockdown of Dppa2 caused the most severe teo-cell block and the lowest blastocyst development.

In mice, ZGA occurs at the early two-cell stage [14]. The results described above showed that Dux, Dppa2 and Dppa4 knockdown mainly resulted in two-cell arrest. We speculated that Dux, Dppa2, and Dppa4 may play an important role in the process of ZGA. To verify this hypothesis, eight typical ZGA genes, namely, Gm20767, MuERV-L, Tcstv1, Tdpoz4, Usp17la, Zfp352, Zscan4 and Zscan4d, were selected [15,16]. The qPCR results showed that the expressions of these eight genes were decreased significantly when Dux, Dppa2, and Dppa4 were jointly knocked down (Figure 1D). Subsequently, we injected the embryos with Dux&Dppa2-siRNA, Dppa2&Dppa4-siRNA, and Dux&Dppa4-siRNA. The expression of ZGA genes in the Dppa2-siRNA injection group was significantly reduced (Figure S1). Subsequently, we injected Dux&Dppa2-siRNA, Dppa2&Dppa4-siRNA, and Dux&Dppa4-siRNA into the embryos. The expression of ZGA genes in the Dppa2-siRNA injection group was significantly reduced (Figure S1). The results suggested that Dppa2 is more closely related to ZGA, corresponding to the most obvious two-cell block phenomenon by Dppa2 knockdown.

### 2.2. ScRNA-Seq Analysis of Dppa2 Knockdown Embryos

**Figure 2:**
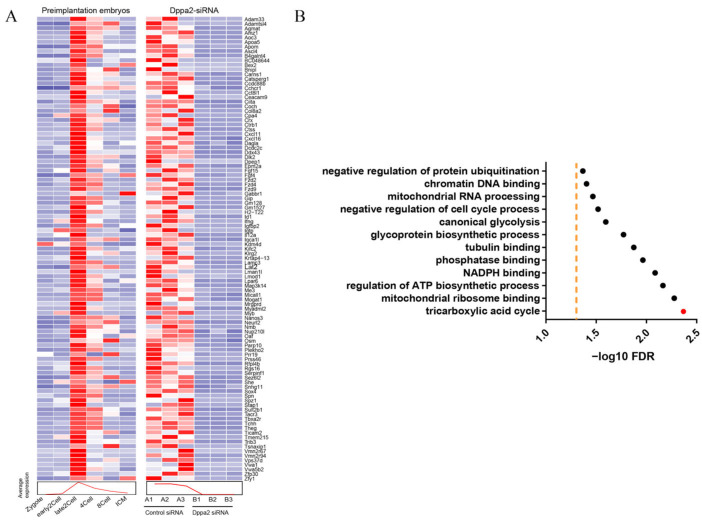
ScRNA-seq analysis of Dppa2 knockdown embryos. (**A**) Comparative analysis of ZGA gene expressions in embryos at different stages. (**B**) Enrichment analysis of differential genes in Dppa2 knockdown embryos. Black dots: Different biological processes. Red dot: Tricarboxylic acid cycle. Yellow line: A significance threshold. *n* = 3.

In order to further explore the mechanism of Dppa2 during the ZGA process, scRNA-Seq was performed using Dppa2 knockdown embryos. We then compared the differentially expressed genes from these embryos with publicly reported ZGA-specific gene sets. In control embryos, a broad and coordinated activation of ZGA-specific genes was observed, as indicated by the strong expression signals across the heatmap. In contrast, Dppa2 knockdown embryos showed a marked reduction in the expression of these genes, reflecting the impaired or failed activation of the ZGA program. These findings indicate that Dppa2 is critical for initiating ZGA, and its depletion results in widespread transcriptional silencing during this key developmental stage. (Figure 2A). Dppa2 knockdown seriously blocked the ZGA process. Functional classification analysis of the differentially expressed genes in Dppa2 knockdown embryos revealed that significant differences in the genes were those particularly associated with the TCA cycle (Figure 2B). This finding suggests that the abundance of two-cell blockage in embryos after Dppa2 knockdown could be attributed to insufficient ZGA-specific gene activation, which has a cascading effect on the TCA cycle process.

### 2.3. Relationship Between Dppa2 and PDH Position During Embryonic ZGA

In a previous report, the core enzymes in the TCA cycle such as pyruvate dehydrogenase (PDH), pyruvate carboxylase (PCB), citrate synthase (CS), aconitase (ACO), and isocitrate dehydrogenase 3 (IDH3) temporarily entered the nucleus during mouse ZGA [17]. PDH is a pivotal enzyme that catalyzes the oxidative decarboxylation of pyruvate to acetyl-CoA, which is an entry substance of the TCA cycle and provides energy for various biological processes. The TCA cycle is a common pathway for the ultimate metabolism of sugars, lipids, and amino acids. Therefore, in this study, PDH was selected as the prototype enzyme of the core TCA cycle to evaluate the relationship between Dppa2 expression and PDH subcellular localization during ZGA.

**Figure 3:**
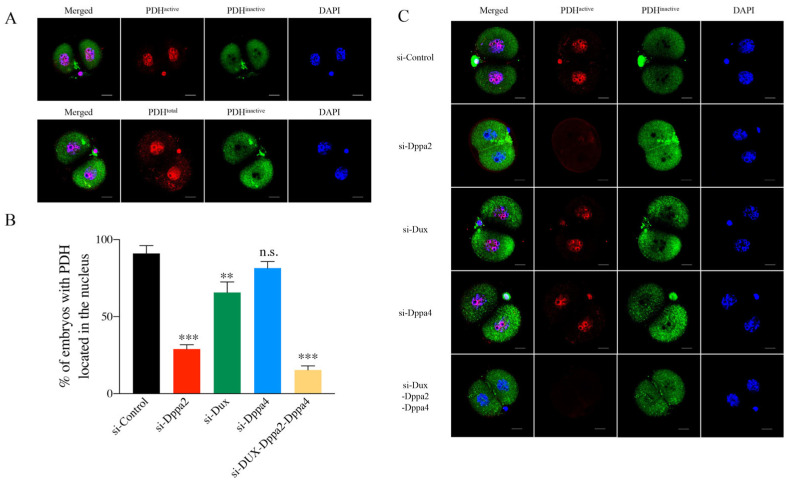
PDH localization in siRNA-injected embryos. (**A**) Distribution of PDH in normal embryos. (**B**) Percentage of embryos with PDH located in nuclei after different gene knockdown. Two-cell embryos were collected 46 h after hCG injection. scale bar, 20 µm. ** *p* < 0.01, *** *p* < 0.001, n.s., not significant. *n* = 3. (**C**) Distribution of PDH in si-injected embryos.

To distinguish the subcellular localization of PDH, we utilized three distinct antibodies to target total pyruvate dehydrogenase (PDH total), active pyruvate dehydrogenase (PDH active), and inactive pyruvate dehydrogenase (PDH inactive). The immunofluorescence staining of the control embryos showed that PDH total was always present in both the cytoplasm and nucleus, while PDH active was only localized in the nucleus, and PDH inactive was predominantly localized in the cytoplasm (Figure 3A).

Next, we tried to determine the nuclear localization of PDH in embryos after Dppa2 knockdown. Late two-cell embryos derived from 46 h post-hCG injection were collected, and immunofluorescence assays were conducted on them to determine the location of PDH [18]. The results indicated that 91.01% of the control embryos injected with Control-siRNA had a nuclear PDH position (Figure 3B,C and Table S2).

However, the individual knockdown of Dppa2 significantly impaired PDH nuclear localization, with only 28.96% of embryos exhibiting PDH in the nucleus (Figure 3B,C and Table S2). Likewise, Dux knockdown also reduced PDH nuclear localization, though to a lesser extent, with 65.68% of embryos showing nuclear PDH. In contrast, Dppa4 knockdown did not affect PDH nuclear localization, as 81.54% of embryos retained PDH in the nucleus, a level comparable to the control group (*p* > 0.5) (Figure 3B,C and Table S2). Moreover, the co-knockdown of Dux, Dppa2, and Dppa4 further reduced PDH nuclear localization, with only 15.41% of embryos showing PDH in the nucleus (Figure 3B,C and Table S2). These results indicate that Dppa2 is a key factor for PDH nuclear localization during early embryogenesis, and that its depletion, as well as the depletion of Dux, significantly impairs this process, potentially affecting energy metabolism. Furthermore, the individual knockdown of Dppa2 had a more pronounced effect on the TCA cycle than Dux or Dppa4 knockdown. These findings strongly link Dppa2 expression to embryonic metabolism and provide valuable insights into the developmental processes of early embryogenesis.

### 2.4. Effect of Dppa2 Overexpression on PDH Localization in Embryos

The rigorous Dppa2 knockdown experiments and careful analyses of single-cell RNA sequencing has proved that Dppa2 is crucially involved in the complex and multi-step process of ZGA. Our findings also indicate that Dppa2 has a significant influence on the localization pattern of PDH, which may have a profound impact on the energy metabolic pathways of embryonic development. To further substantiate our conjecture, we designed a Dppa2 overexpression mouse model to examine the intricate relationships between Dppa2 and core TCA enzymes.

**Figure 4:**
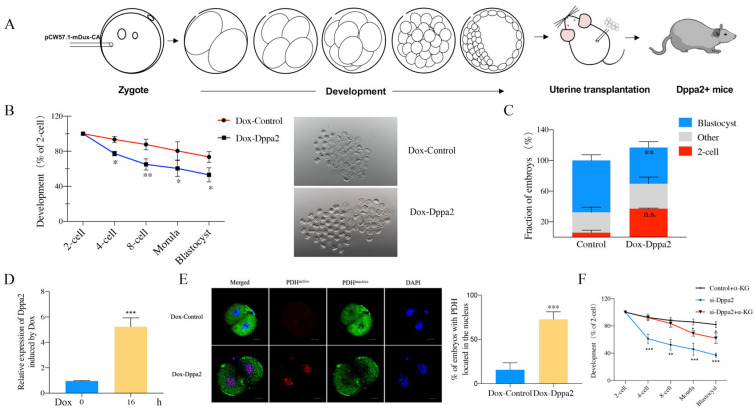
Developmental rate of embryos after Dppa2 overexpression and PDH distribution in two-cell embryos. (**A**) Schematic diagram of Dppa2 overexpression mouse production. (**B**) Developmental rates of Dppa2-overexpressing embryos. * *p* <0.05, ** *p* < 0.01. n=3. (**C**) Embryonic development arrestment after Dppa2 overexpression. ** *p* < 0.01, n.s., not significant, n=3. (**D**) Relative expression levels of Dppa2 overexpression. (**E**) PDH immunostaining distribution in embryos after Dppa2 overexpression. Percentages of PDH localized in nuclei of Dppa2-overexpressing embryos. Two-cell embryos were collected 36 h after hCG injection. *** *p* < 0.001. *n* = 3. (**F**) Effect of α-KG on embryo developmental rate. * *p* <0.05, ** *p* < 0.01, *** *p* < 0.001. *n* = 3.

To investigate the effects of Dppa2 overexpression on early embryonic development, we generated transgenic mice harboring a doxycycline (Dox)-inducible Dppa2 expression vector utilizing a Tet-on system. Linearized vectors were microinjected into pronuclei, and Dppa2-positive (Dppa2+) founder mice were identified (Figure 4A). Subsequently, the developmental competence of Dppa2-overexpressing embryos was assessed. Notably, the blastocyst formation rate was significantly reduced in Dox-treated embryos (53.12 ± 8.05%) compared to controls (73.55 ± 6.12, *p* < 0.05) (Figure 4B and Table S3). Furthermore, 20.15 ± 2.70% of Dox-treated embryos exhibited two-cell arrest (Figure 4C). These findings demonstrate that the Dox-induced overexpression of Dppa2 leads to a significant decrease in blastocyst development and a substantial increase in two-cell arrest.

To induce Dppa2 overexpression, Dppa2+ female mice were superovulated and mated with Dppa2+ males. Fertilized zygotes were collected and cultured in vitro in the presence of Dox, initiating induction at the one-cell stage, specifically between 12 and 36 h post-hCG administration. Quantitative RT-PCR confirmed a significant increase in Dppa2 mRNA levels in Dox-treated two-cell embryos compared to controls after 16 h of induction, validating successful overexpression (Figure 4D). In view of the changes in PDH position in the embryos after Dppa2 deletion, we attempted to further investigate the relationship between Dppa2 and PDH position. We first detected the PDH localization in early two-cell embryos (36 h post-hCG injection). As expected, control embryos exhibited minimal nuclear PDH at this stage, contrasting with the substantial nuclear PDH observed in late two-cell embryos (46 h post-hCG injection). Dppa2 overexpression was induced at the one-cell stage, and early two-cell (36 h post-hCG injection) embryos were subsequently collected for immunofluorescence analysis of PDH localization. Significantly, early two-cell embryos overexpressing Dppa2 exhibited a marked increase in nuclear PDH localization (72.75 ± 8.49%) compared to controls (15.60 ± 7.92%, *p* < 0.001). (Figure 4E and Table S4). Despite embryo collection occurring approximately 10 h earlier than the typical time of PDH nuclear entry, a marked increase in nuclear PDH was evident in Dppa2-overexpressing embryos. These findings strongly suggest that Dppa2 overexpression promotes the precocious nuclear translocation of PDH, thereby reinforcing a direct regulatory link between Dppa2 and PDH subcellular localization. These results confirm the existence of a regulatory relationship between Dppa2 and the TCA cycle. Furthermore, we demonstrated that supplementation with α-ketoglutarate (α-KG), a key metabolite of the TCA cycle, could rescue the embryonic developmental arrest caused by ZGA blockage following Dppa2 knockdown. Specifically, the blastocyst formation rate increased from 37.10 ± 3.01% to 62.01 ± 7.78% after α-KG supplementation (*p* < 0.01) (Figure 4F and Table S5). These findings further support the notion that there is a close regulatory connection between ZGA and TCA cycle activity.

## 3. Discussion

The onset of ZGA is a critical event in embryonic development. Any slight error or disruption during ZGA can result in abnormal embryo development or the termination of development. The current understanding of the regulatory mechanisms of ZGA processes in mammals is limited [19,20,21]. Previously, Dux was reported as a crucial activator of ZGA in mice [5,22,23]. However, when Dux was knocked out, homozygous knockdown mice were still generated, while the number of offspring was halved compared to normal mice [7]. This demonstrates that Dux deficiency has some impact on embryonic development but is not indispensable. The present results are consistent with this finding. The knockout of Dux in mouse embryos resulted in significant two-cell developmental block and significantly reduced embryo development. However, 43.81% of the embryos still could develop to blastocysts.

Alongside Dux, Dppa2 and Dppa4 transcripts are present in mouse zygotes and act as key upstream mediators for ZGA. These genes are in the regulatory region of the embryonic genome and are affected by DNA methylation and histone modifications [10]. During ZGA, Dppa2 and Dppa4 bind to the promoters of ZGA-related genes, activate their transcription, and inhibit repressor recruitment [24]. Our results show that the knockdown of Dux, Dppa2, and Dppa4 significantly affected ZGA in mouse embryos, leading to decreased embryo development. Dppa2 knockdown resulted in the most significant reduction in embryo development, while the knockdown of Dppa4 had a minor effect. Dppa2 and Dppa4 are homologous genes related to developmental pluripotency, and although their expressions are similar during embryo development, there are regulatory differences [24]. The overexpression of Dppa2 activates ZGA more efficiently, while the overexpression of Dppa4 does not directly affect ZGA but increases Dppa2 expression [9,25,26]. The complex interaction between Dppa2 and Dppa4 and their functional differences may explain the significant difference in the rates of embryo development after their deletion.

Interestingly, even after Dppa2 knockdown, approximately 33.20% of the embryos could still develop to the blastocyst stage, and the co-knockdown of Dux, Dppa2, and Dppa4 still allowed 19.78% of the embryos to form blastocysts. These findings imply that, in addition to Dux, Dppa2, and Dppa4, other regulatory factors are likely involved in orchestrating ZGA and embryonic development. The single-cell RNA sequencing of Dppa2 knockdown embryos revealed a global reduction in ZGA-associated gene expression, further confirming the crucial role of Dppa2 in ZGA regulation. Remarkably, our scRNA-seq data also revealed that Dppa2 knockdown affected genes related to the tricarboxylic acid (TCA) cycle, suggesting a potential link between Dppa2-mediated transcriptional activation and metabolic regulation during early development.

During preimplantation development in mammals, the embryos expend a small amount of energy to complete the normal development process [27]. In bovine, embryos develop to the morula stage using the energy provided by pyruvate oxidation. During the very early stage, embryos need little energy, and pyruvate oxidation becomes the first choice to supply energy for embryo development [28]. In mice, the glucose metabolism level of preimplantation embryos is far lower than that of somatic cells. As the main metabolic pathway, the TCA cycle provides energy for embryos, and citric acid in embryo metabolites is very rich [29]. Studies have shown that PDH, PCB, CS, ACO, and IDH3 in the TCA cycle enter the nuclei during the ZGA period in mouse embryos [17]. These enzymes, called class I enzymes, are involved in the first half of the TCA cycle and are activated in the nuclei [17]. The transition of class I enzymes from the cytoplasm to the nucleus plays a crucial role in ZGA and facilitates the occurrence of epigenetic modification [17]. Enzymes that act on mitochondria in the TCA cycle are called class II enzymes, but they do not display similar position changes. In addition, the accumulation of succinic acids and fumaric acids in class II enzyme metabolites is detrimental to embryonic ZGA [17]. During glycolysis, glucose is converted to pyruvate under anaerobic conditions and then converted to acetyl-CoA via PDH, entering the TCA cycle [30]. As an energy source, pyruvic acids play an important role during embryo development [31]. Therefore, PDH was selected as the main research object of TCA cycle ribozymes in this experiment.

**Figure 5:**
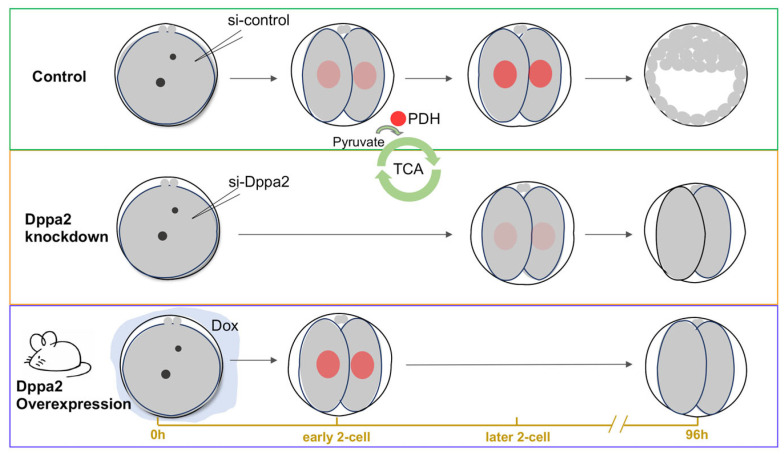
Dppa2 is a key regulator of PDH localization within the TCA cycle during ZGA.

The present results show evidence that ZGA is closely related to energy metabolism, and Dppa2 plays an important role in regulating both ZGA and energy metabolism. The knockdown of Dux, Dppa2, and Dppa4 genes led to a significant decrease in the proportion of embryos with a nuclear localization of PDH, which indicates that PDH nuclear entry is blocked when ZGA does not occur. The knockdown of Dppa2 had the greatest impact on the entry of PDH, indicating that it has the strongest ability to regulate PDH position changes. To further explore the role of Dppa2, we generated Dppa2-overexpressing transgenic mice. Consistent with the knockdown results, the overexpression of Dppa2 also impaired embryonic development, reducing blastocyst formation rates and increasing two-cell developmental arrest. Notably, Dppa2 overexpression induced the premature nuclear localization of PDH, occurring earlier than the typical ZGA period. These results suggest that both insufficient and excessive Dppa2 expression disrupts the finely tuned spatiotemporal regulation of ZGA and metabolic remodeling, emphasizing the need for balanced Dppa2 expression during early development (Figure 5).

We further explored whether supplementation with metabolic intermediates could rescue the developmental defects caused by Dppa2 knockdown. The addition of α-KG, a key metabolite in the TCA cycle, effectively alleviated the developmental arrest in Dppa2-deficient embryos, significantly improving blastocyst formation rates. However, the developmental potential of α-KG-treated embryos did not fully recover to the level of control embryos. We speculate that the absence of Dppa2 leads to the impaired nuclear entry of upstream metabolic enzymes, resulting in compromised TCA cycle activity and energy deficiency during ZGA. Although α-KG supplementation can partially compensate for this metabolic imbalance, it cannot restore the transcriptional regulatory functions of Dppa2, highlighting the complex interplay between transcriptional and metabolic regulation during early embryogenesis.

Indeed, the findings of this study provide valuable insights into the complex regulatory mechanisms of embryonic development and metabolic regulation. Dux, Dppa2, and Dppa4 are key players in ZGA, and PDH is involved in energy metabolism, which sheds light on the intricate interplay between genetic and metabolic processes during embryonic development. We speculate that Dppa2 may influence PDH nuclear localization indirectly by modulating chromatin accessibility, facilitating the recruitment of nuclear import machinery, or affecting the transcription of metabolic regulators involved in TCA cycle enzyme translocation. Alternatively, Dppa2 may interact with specific epigenetic factors or nuclear structural components that promote the temporary nuclear entry of metabolic enzymes during ZGA. However, the precise molecular mechanism by which Dppa2 coordinates transcriptional and metabolic regulation requires further investigation in future studies.

## 4. Materials and Methods

### 4.1. Animals

All animal studies were approved by the Animal Care and Use Committee of Inner Mongolia University. All procedures were carried out in strict accordance with the recommendations made in the Guide for the Care and Use of Laboratory Animals of the National Veterinary and Quarantine Service. BDF1 (C57BL/6N 3 DBA/2), CD1, and Kunming mice were purchased from Laboratory Animal Research Center (Inner Mongolia University, China) or Vital River Laboratories (Beijing, China).

### 4.2. Superovulation and Embryo Culture

Female BDF1 mice aged 8 weeks were superovulated by an intraperitoneal injection of 10 IU pregnant mare serum gonadotropin (PMSG, Ningbo second hormone factory, Ningbo, China), and then injected with 10 IU human chorionic mgonadotropin (hCG, Ningbo second hormone factory, Ningbo, China) 48 h after PMSG injection. After hCG injection, the females were mated with BDF1 males. The mice were killed by cervical dislocation 18 h post-hCG, and successful mating was confirmed by the presence of vaginal plugs. The fertilized 1-cell zygotes were collected from oviduct ampullae and treated with 1% hyaluronidase (Sigma, St. Louis, MI, USA) to disperse the surrounding cumulus cells. The zygotes were then cultured in KSOM medium (Sigma, St. Louis, MI, USA) under 37 °C in 5% CO_2_ in air.

### 4.3. siRNA Construction and Microinjection in Oocytes

All siRNAs involved in this experiment are purchased from Gemma Gene (Table S6). The sequences are shown in Table S6. As previously described [32], 8 pl of siRNA was microinjected into the cytoplasm of the denuded oocytes using a piezo-operated blunt-end micropipette (3–5 lm internal diameter). After injection, the oocytes were kept at room temperature for 30 min and then moved into the incubator.

### 4.4. RNA Extraction and RT–qPCR

Twenty embryos were collected for total RNA extraction using a Pico-Pure RNA Isolation Kit (Thermo Fisher Scientific) according to the manufacturer’s instructions. Reverse transcription was performed using a SuperScript III kit (Thermo Fisher Scientific, Waltham, MA, USA). The reverse transcription of cDNA was carried out using a PrimeScript RT Reagent Kit (TaKaRa, Tokyo, Japan). Real-time quantitative RT-PCR was performed using an SYBR Premix Ex Taq (Takara, Kusatsu, Japan). Signals were detected with an Applied Bio-systems 7500 Real-Time PCR System (Applied Biosystems, Waltham, MA, USA). The relative expression of target genes was analyzed by the 2^−ΔΔCT^ method. Primers are shown in Supplemental Table S7.

### 4.5. Immunofluorescence Staining

The embryos were fixed in 4% paraformaldehyde (PFA) overnight at 4 °C, and then permeabilized for 30 min in PBS with 0.4% (*vol.*/*vol*) TritonX-100 (PBST), blocked in PBS containing 3% bovine serum albumin for 1 h at 37 °C, and incubated with the primary antibodies in PBS 0.3% bovine serum albumin overnight at 4 °C. Subsequently, the embryos were incubated for 1h with the primary antibodies at 37 °C. The appropriate secondary antibodies were conjugated with Alexa Fluor 594 and Alexa Fluor 488 (Thermo, Waltham, MA, USA) for 1h at 37 °C. Then, the treated embryos were laced in 5 μL DAPI (Thermo, Waltham, MA, USA). All samples were observed under a laser scanning microscope (Nikon, Tokyo, Japan). Table S8 shows all the antibodies used in this study.

### 4.6. Generation of Transgenic Mice

The pCW57.1-mDux-CA vector (Addgene 99284) was digested with both 5’Nhe I and 3’ Sal I. The Dux fragments and the carrier skeleton were separated by agarose gel electrophoresis, and the carrier skeleton was recovered by gel cutting. Finally, the mouse Dppa2 sequence, synthesized by Shanghai Biotechnology, was connected to the pCW57.1-mDux-CA vector skeleton through homologous recombination to construct the pCW57.1-mDppa2-CA vector. Then, the modified vectors were linearized with enzyme. By using CRISPR-Cas9-mediated gene knock-in at the Rosa26 locus, we co-injected the CRISPR/Cas9 plasmid and the linearized donor fragment into fertilized zygotes to generate positive homozygous mice [33]. After injection, the embryos were cultured in KSOM-AA medium for 4 h and then transferred to the pseudopregnant female mice. In order to identify the founder, the tail tip of young mice was used for DNA extraction, and positive mice were identified by PCR.

### 4.7. Dppa2-Overexpressing Embryo Collection

To generate embryos with induced Dppa2 overexpression, heterozygous dox-inducible Dppa2 transgenic (Dppa2+) female mice were superovulated and mated with heterozygous Dppa2+ male mice. Zygotes were collected from the oviductal ampullae of females 18 h post-hCG injection, as confirmed by the presence of vaginal plugs. Dppa2 overexpression was induced by supplementing the KSOM culture medium with 2 μg/mL doxycycline. Early 2-cell embryos of Dppa2 overexpression were collected 16 h after the initiation of doxycycline treatment.

### 4.8. Low-Input ScRNA-Seq

Twenty high-quality zygotes, previously microinjected with either control siRNA or Dppa2-specific siRNA, and with the zona pellucida removed, were collected and subsequently lysed in cell lysis buffer. The resulting lysates were immediately flash-frozen in liquid nitrogen and stored at −80 °C. RNA-seq libraries were generated using the Smart-Seq2 protocol and subsequently sequenced on an Illumina HiSeq platform by An Nuoyouda Gene Tech Co., Ltd. (Beijing, China). The quality and quantity of the constructed DNA libraries were assessed using a Qubit™ 4 Fluorometer and a Bioanalyzer 2100 (Invitrogen), respectively.

### 4.9. RNA-Seq Analysis

Paired-end raw reads generated from Smart-Seq2 sequencing underwent quality control using Fastp (version 0.23.2) to produce high-quality clean reads. These clean reads were then utilized for subsequent analyses. Alignment to the mouse genome (mm10) was performed using HISAT2 (version 2.2.1) with default parameters. Uniquely mapped reads were assembled into exon features, guided by the reference annotation (GENCODE vM23) obtained from the University of California Santa Cruz (UCSC). Gene expression levels were quantified as reads per kilobase million (RPKM). Differential gene expression analysis was conducted using the DESeq2 package within R software (version 4.3.1). Genes demonstrating a fold change of ≥4 and a padj of ≤0.05 were defined as differentially expressed. Gene Ontology (GO) enrichment analysis was performed using the clusterProfiler package in R, with a significance threshold of *p* ≤ 0.05. The raw sequence data reported in this paper were deposited in NCBI (ID: PRJNA1231683); open access is available at https://www.ncbi.nlm.nih.gov/geo/info/linking.html (accessed on 5 March 2025). The publicly available datasets used in this work were from NCBI GEO accession number GSE71434 (RNA-seq).

## 5. Conclusions

The key activators of ZGA, Dux, Dppa2, and Dppa4 were closely related to preimplantation embryonic development in mice, and Dppa2 had the most significant effect on embryonic development. After Dppa2 knockdown, the embryos underwent severe two-cell arrest, resulting in a blastocyst development rate from 80.36% to 33.20% (*p* < 0.001), and a significant decrease in the proportion of PDH nucleation during ZGA (91.01% vs. 28.96%, *p* < 0.001). When Dppa2 is overexpressed, PDH can be induced to enter the nucleus early. It is inferred that Dppa2 plays a key role in the activation of ZGA, which directly determines the localization of TCA core enzymes in early embryos and affects early embryonic development.

## Data Availability

The data generated and analyzed during this study are available upon reasonable request from the corresponding author.

## Supplementary Materials

The following supporting information can be downloaded at: https://www.mdpi.com/article/10.3390/ijms26073436/s1.
