# Supplementary material for: Dppa2 Promotes Early Embryo Development Through Regulating PDH Expression Pattern During Zygotic Genome Activation
Source: Int J Mol Sci. 2025 Apr 6;26(7):3436. doi: 10.3390/ijms26073436 (PMC11989748; doi:10.3390/ijms26073436)
Supplement: Supplementary file 1 [file ijms-26-03436-s001.zip › Supplemental Figure S1.pdf]

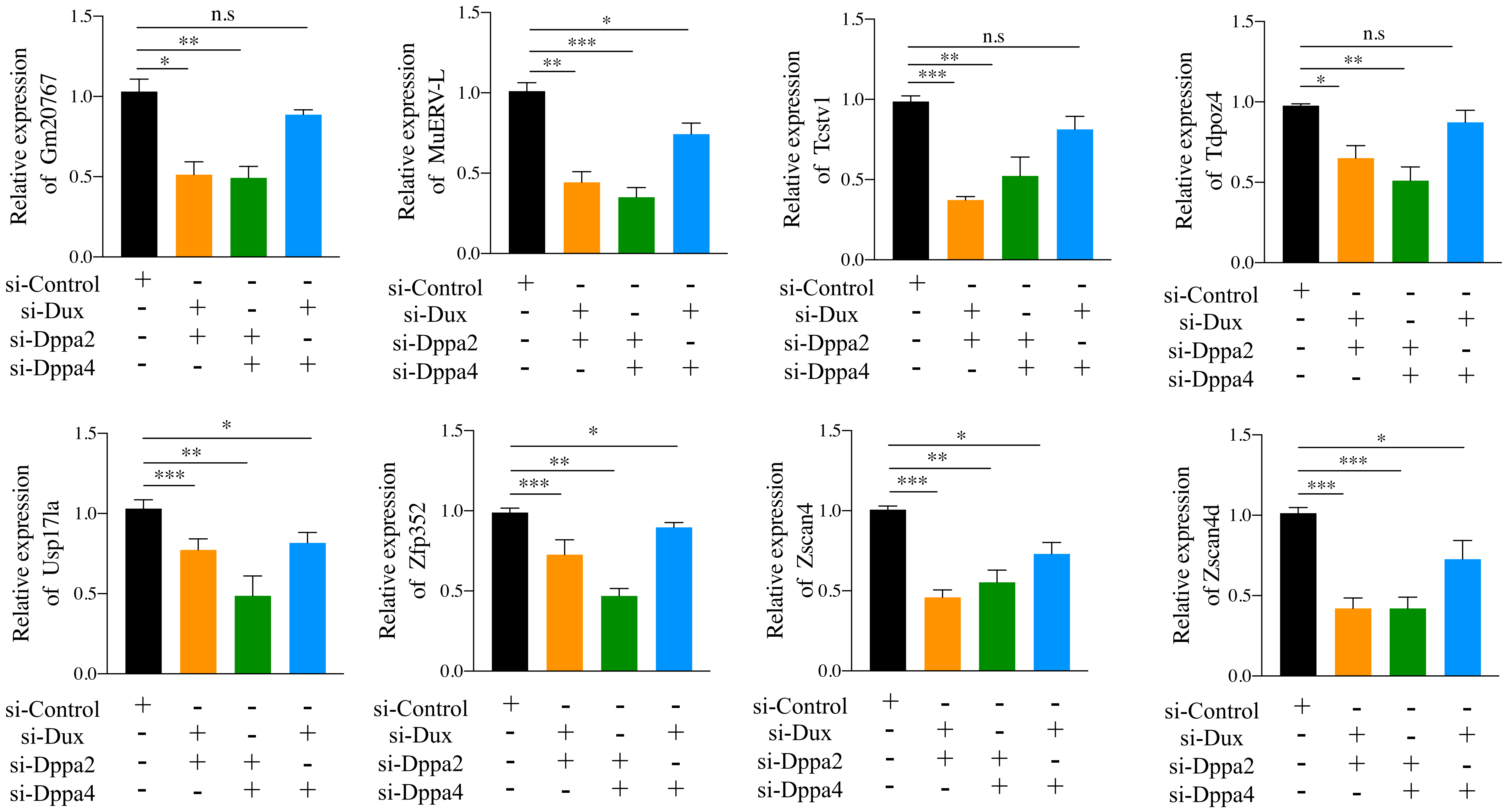

Relative expression of Gm20767
1.5
1.0
0.5
0.0
n.s
**
*
Relative expression of MuERV-L
*
***
**
Relative expression of Tcstv1
n.s
**
***
Relative expression of Tdpoz4
n.s
**
*
Relative expression of Usp17la
*
**
***
Relative expression of Zfp352
*
**
***
Relative expression of Zscan4
*
**
***
Relative expression of Zscan4d
*
***
***
si-Control + - - -
si-Dux - + - +
si-Dppa2 - + + -
si-Dppa4 - - + +
