# Supplementary material for: Dppa2 Promotes Early Embryo Development Through Regulating PDH Expression Pattern During Zygotic Genome Activation
Source: Int J Mol Sci. 2025 Apr 6;26(7):3436. doi: 10.3390/ijms26073436 (PMC11989748; doi:10.3390/ijms26073436)
Supplement: Supplementary file 1 [file ijms-26-03436-s001.zip › Table.pdf]

Table S1. Developmental rates of embryos derived using different siRNA

Group	No. of replicates	No. of cultured	No. of 2-cell (% of cultures)	No. of 4-cell (% of cleaved)	No. of 8-cell (% of cleaved)	No. of morula (% of cleaved)	No. of blastocyst (% of cleaved)
Si-Control	3	166	94.18±1.49	91.81±5.43	86.71±9.24	83.51±10.49	80.36±9.81
Si-Dppa2	3	175	94.47±3.21	52.31±14.58***	46.85±14.81***	39.62±11.97***	33.20±4.10***
Si-Dppa4	3	195	94.94±2.01	74.87±8.18*	66.31±5.17*	61.43±5.42*	58.15±7.09*
Si-Dux	3	189	97.85±1.03	59.90±4.23***	53.47±1.59***	46.46±1.80***	43.81±2.69***
Si-Dppa2 -Dppa4 -Dux	3	178	92.68±0.60	34.29±6.15***	28.28±5.00***	23.35±3.74***	19.78±5.05***

* *P* <0.05, ****P* <0.001, by two-way ANOVA.

Table S2. The birth of F0 generation mice

No. of Zygotes	No. of 2-cell (% of 1-cell)	No. of blastocyst (% of 2-cell)	Embryos Transferred stage	No. of embryos Transferred (ET) per recipient	No. of pups at full-term	No. of positive pups (% of pups)
154	147 (95.45%)	106 (72.1%)	blastocyst	15	12	9 (75%)

Table S3. Developmental rates of embryos derived Dppa2 over-expression

Group	No. of replicates	No. of cultured	No. of 2-cell (% of cultures)	No. of 4-cell (% of cleaved)	No. of 8-cell (% of cleaved)	No. of morula (% of cleaved)	No. of blastocyst (% of cleaved)
Dox-Control	3	131	91.78±2.82	93.47±3.57	87.76±6.00	80.46±10.44	73.55±6.12
Dox-Dppa2	3	170	88.92±1.67	77.37±2.64*	65.03±6.45**	60.48±9.11*	53.12±8.05*

* *P* <0.05, ***P* <0.01, by two-way ANOVA.

Table S4. Statistics of PDH into nuclei after different gene knockdown

Groups	Numbers	Collection time（after hCG）	% Of PDH located in nucleus the total number of embryos
si-Control	69	46 h	91.01 ± 5.15%
si-Dux-Dppa2-Dppa4	65	46 h	15.41 ± 2.59%***
si-Dux	67	46 h	65.68± 6.83%**
si-Dppa2	66	46 h	28.96 ± 2.86%***
si-Dppa4	70	46 h	81.54± 4.27%

** P <0.01, ***P <0.001, by one-way ANOVA.

Table S5. Statistics of PDH into nuclei after Dppa2 over-expression

Groups	Numbers	Collection time（after hCG）	% of PDH located in nucleus the total number of embryos
Dox-Control	66	36 h	15.60 ± 7.92%
Dox-Dppa2	63	36 h	72.75 ± 8.49%***

***P <0.001, by two-tailed Student's t-test.

Table S6. The siRNA sequences used in this study

Gene	siRNA sequence （5′ - 3′）
Dux-siRNA	Sense- GUAAAUUCGGGCAUGGAUATT<br>Antisense- UAUCCAUGCCCGAAUUUACTT
Dppa2-siRNA	Sense- CCUGUUACCGGCCUGUAUUTT<br>Antisense- AAUACAGGCCGGUAACAGGTT
Dppa4-siRNA	Sense- CAGCACAACGGGCGUCAUATT<br>Antisense- UAUGACGCCCGUUGUGCUGTT
Control-siRNA	Sense- UCUUAAUCGCGUAUAAGGCTT<br>Antisense- GCCUUAUACGCGAUUAAGATT

Table S7. A tabulation of primer sequences

Primers	Applications	Sequence（5′ - 3′）
Dppa2	PCR	Forward- TCGTTTCAGACCCACCT<br>Reverse- GCCATCCGTACTCAAGTTA
Dux	qPCR	Forward- AACCCACGACCAGGCTTTG<br>Reverse- CCGAGCTCTTCGGTTTTGAA
Dppa2	qPCR	Forward- AGCCGTGCAAAGAAAAATGC<br>Reverse- TATTCTTCCATTCCCTTTAGATCAGAGT
Dppa4	qPCR	Forward- TGAACCTGATTCACCGAGATGT<br>Reverse- TGCTGCTCACTCGTTTCTTCTG
MuERV-L	qPCR	Forward- ATCTCCTGGCACCTGGTATG<br>Reverse- AGAAGAAGGCATTTGCCAGA
Zscan4d	qPCR	Forward- GCAGATGCCAGTAGACACCA<br>Reverse- GGCATCAAGAGGGAATTGAA
Tcstv1	qPCR	Forward- GGATCCCTGAAGGTAAATCCTC<br>Reverse- AACCATCCATCCTCAGGAAC
Usp17la	qPCR	Forward- TTTGTAGACACGGTGGTTGC<br>Reverse- GGGAGCAGAAGGAAGTTTTTC
Zscan4	qPCR	Forward- GAGATTCATGGAGAGTCTGACTGATGAGTG<br>Reverse- GCTGTTGTTTCAAAAGCTTGATGACTTC
Zfp352	qPCR	Forward- AAAGCCTTGATCCTCAGGTG<br>Reverse- GCCGAAGAGTTTTTCTGAGG
Tdpoz4	qPCR	Forward- ACCCAAGACCTGCAATCAAG<br>Reverse- ATTCATGGCCAGCTACCAAC
Gm20767	qPCR	Forward- TGCTTCCTATCCAGCTCTTG<br>Reverse- CGGAAAAGGACTGCATCATC

Gapdh	qPCR	Forward- GTGGCAAAGTGGAGATTGTTG<br>Reverse- CTCCTGGAAGATGGTGATGG

Table S8. Antibodies used in this study

Antibodies	Company	Cat.
Rabbit anti-Phospho-PDH	Abcam	ab92696
Mouse anti-PDH（active）	Abcam	ab110334
Mouse anti-PDH（total）	Santa Cruz	sc-377092
